# MantaID: a machine learning–based tool to automate the identification of biological database IDs

**DOI:** 10.1093/database/baad028

**Published:** 2023-05-09

**Authors:** Zhengpeng Zeng, Jiamin Hu, Miyuan Cao, Bingbing Li, Xiting Wang, Feng Yu, Longfei Mao

**Affiliations:** Department of Pharmacy, College of Biology, Hunan University, No. 27, Tianma Road, Changsha 410082, P.R. China; Department of Pharmacy, College of Biology, Hunan University, No. 27, Tianma Road, Changsha 410082, P.R. China; Department of Pharmacy, College of Biology, Hunan University, No. 27, Tianma Road, Changsha 410082, P.R. China; Department of Pharmacy, College of Biology, Hunan University, No. 27, Tianma Road, Changsha 410082, P.R. China; Department of Pharmacy, College of Biology, Hunan University, No. 27, Tianma Road, Changsha 410082, P.R. China; State Key Laboratory of Chemo/Biosensing and Chemometrics, College of Biology, Hunan Key Laboratory of Plant Functional Genomics and Developmental Regulation, Hunan University, No. 27, Tianma Road, Changsha 410082, P.R. China; State Key Laboratory of Hybrid Rice, Hunan Agricultural Biotechnology Research Institute, Hunan Academy of Agricultural Sciences, No. 27, Tianma Road, Changsha 410125, P.R. China; Department of Pharmacy, College of Biology, Hunan University, No. 27, Tianma Road, Changsha 410082, P.R. China

## Abstract

The number of biological databases is growing rapidly, but different databases use different identifiers (IDs) to refer to the same biological entity. The inconsistency in IDs impedes the integration of various types of biological data. To resolve the problem, we developed MantaID, a data-driven, machine learning–based approach that automates identifying IDs on a large scale. The MantaID model’s prediction accuracy was proven to be 99%, and it correctly and effectively predicted 100,000 ID entries within 2 min. MantaID supports the discovery and exploitation of ID from large quantities of databases (e.g. up to 542 biological databases). An easy-to-use freely available open-source software R package, a user-friendly web application and application programming interfaces were also developed for MantaID to improve applicability. To our knowledge, MantaID is the first tool that enables an automatic, quick, accurate and comprehensive identification of large quantities of IDs and can therefore be used as a starting point to facilitate the complex assimilation and aggregation of biological data across diverse databases.

Key points

- MantaID is a data-driven, machine learning–based method that automatically identifies IDs with high accuracy and efficiency and at a large scale.
- The accuracy of MantaID is confirmed using common statistical metrics.
- A novel metric method is devised to verify the performance of MantaID.
- MantaID is implemented as an R package, as well as a web app and application programming interface for easy use.

## Introduction

Identifiers (IDs) are used in databases to index and code biological data. As of January 2022, there were 1645 databases and approximately 1700 registered ID nomenclatures (1, 2). IDs are required for simple access to biological data and for facilitating cross-referencing between databases. However, each database has its own representation and a set of ID numbers for identifying biological components (3–9), indicating that IDs from different databases may overlap, that is, the same biological entity may have various IDs (10). For example, a molecule can possess both an Entrez ID (11) and an Ensembl ID (12, 13); Ring Finger Protein 180 is represented by a variety of IDs, including HGNC ID 27752, an Entrez ID 285671, an ENSG00000164197 Ensembl ID, OMIM ID 616015, etc. We observed that different databases tend to employ distinct naming conventions. The first three digits of ID in the Ensembl database, for example, begin with ‘ENS’; the fourth digit of ‘G’ is for gene, ‘T’ is for transcript and ‘P’ is for protein; and then the ID ends with number; in the Entrez gene database, pure numbers are used as gene IDs, beginning with ‘NM’ for transcript number, ‘NP’ for protein number and ‘NR’ for non-coding RNA number; a letter plus a number is used in the UniProt database. In contrast, Kyoto Encyclopedia of Genes and Genomes IDs are composed of a capital letter followed by five digits, while the GO database uses a combination of letters, numbers and underscores. In addition, these IDs may be temporary, which require modification or replacement when new functions for the molecules are revealed. The exchange of information between multiple databases is typically accomplished via mappings between distinct IDs, which has been a cause for concern.

Several ID conversion services, such as UniProt Mapping (14), DAVID (15), BioMart (16), TogoID (17) and GeneToList (18), have been developed to solve this issue. These ID conversion tools enable ID–ID mapping to convert a gene or gene product from one type to another (19). In addition, these tools also implement special features, such as TogoID (17), which can disambiguate and transform IDs. However, they all require previous knowledge of the database to which they belong and are incapable of identifying the IDs in the absence of database names. Therefore, a tool that can automatically construct cross-references between different databases without requiring knowledge of the database names is needed. In this paper, we describe the MantaID tool, which identifies and classifies unknown IDs quickly and precisely by automatically creating ID mappings across multiple databases. This differs from the current ID conversion programs, which rely on ID mappings between databases and only support a limited number of ID types. To our knowledge, MantaID is the first tool for the identification of IDs using machine learning algorithms, which were often used to be applied in various biological applications such as genomic sequence analysis and annotation of proteomics or metabolomics (20).

The computational framework and all the approaches of MantaID are implemented as a software package that handles all the different steps of the model development process and makes it easy to create user-defined ID recognition models by adjusting a few parameters. To demonstrate the usability of MantaID, we have also developed a user-friendly web application that demonstrates the framework approach and workflow for automated ID recognition and enables users to recognize multiple IDs without delving into the model implementation specification. In addition, we provide application programming interface (API) access so that users can launch complex queries programmatically.

## Materials and Methods

For easy reference, we summarize the mathematical notations used throughout this paper in Table 1.

**Table 1.** Mathematical notations and symbols used in this paper

Parameters	Definitions
*D*	Atrain dataframe with label and features columns
*N*	A dataframe for forecasting with feature columns to predict
*K*	A dataframe with feature columns and predict column
${s_{{\rm{max}}}}$	Actual budget for a single hyperparameter configuration
*B*	The total budget
*n*	The number of parameter configurations
*r*	The actual budget for a single hyperparameter configuration
*T*	A grouping of parameter configurations
${n_i}$	Number of bracket configurations
${r_i}$	Resource allocation
*L*	The validation loss of configuration *t*
*R*	Maximum number of resources
$\eta $	The proportion of parameter configurations ‘advances’ to the next round in hyperband tuning
${G_i}$	The Gini index of the ${i_{{\rm{th}}}}$ feature
${\alpha _{{\rm{best}}}}$	Feature that minimizes ${G_i}$
${D_{{\rm{subs}}}}$	Induced sub-datasets from $D$ divided by ${\alpha _{{\rm{best}}}}$
${{\rm{Z}}^{\rm{*}}}$	*D* bootstrap samples
${\rm{Tre}}{{\rm{e}}_{\rm{b}}}{\rm{/Tre}}{{\rm{e}}_{\rm{t}}}$	A weak tree learner
${e_{\rm{b}}}$	The rate of out-of-bag (oob) error
${F_{\rm{b}}}$	A small subset of features
${\rm{Forest}}$	A strong learner made up of weak tree learners
${g_{ti}}$	The *i*th node’s first derivative in round *t*
${h_{ti}}$	The *i*th node’s second derivative in round *t*
${G_t}$	The sum of the first derivatives
${H_t}$	The sum of the second derivatives
${G_{\rm{L}}}$	The sum of the left subtree’s first derivatives
${H_{\rm{R}}}$	The sum of the right subtree’s second derivatives
${G_{\rm{R}}}$	The sum of the right subtree’s first derivatives
${H_{\rm{L}}}$	The sum of the second derivatives of the left subtree
$\gamma $	The regularization coefficient governs the number of leaf nodes’ complexity
$\lambda $	Regularization coefficients that govern the L1–L2 mix
${O_j}$	The value of neuron unit output
${w_{ij}}$	Layer *i* and layer *j* weight matrix
${\theta _i}$	The bias of the ${i_{{\rm{th}}}}$ neuron

### MantaID framework

**Figure 1.**
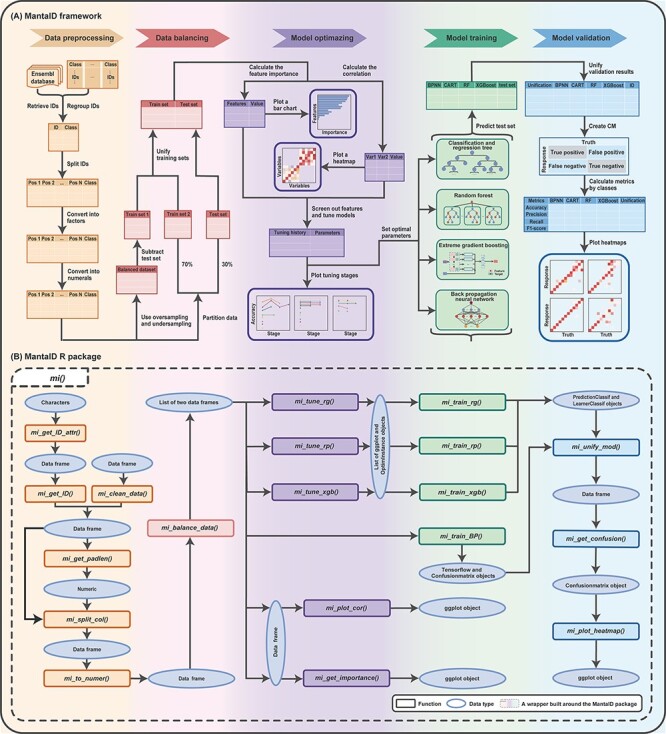
Schematic overview of the MantaID tool. (A) The theoretical framework MantaID. (B) The R package functions of MantaID. The wrapper function created by MantaID; a wrapper function ‘mi()’ is created that is used to group the functionalities of MantaID and can be executed to carry out all the steps of the MantaID workflow in a lazy fashion.

A schematic overview of the MantaID framework can be found in Figure 1A. First, the MantaID workflow begins with a data frame containing ID and class, obtained either by connecting to the public database using the ‘mi_get_ID_attr’ and ‘mi_get_ID’ functions or from other sources after preprocessing such as data frame reshaping and invalid data removal by the ‘mi_clean_data’ function. Next, a data frame containing the ID columns is passed into the ‘mi_get_padlen’ and ‘mi_split_col’ functions, which cut the IDs into a single-character vector of maximum length. After that, it returns a wide data frame in the original order of the samples, containing the location features and class of the IDs. Then, all single-character features are converted into numeric types using a fixed mapping and can be used directly for training by calling the ‘mi_to_numer’ function. Prior to training, the ‘mi_balance_data’ function is developed to oversample and undersample the data using the Synthetic Minority Oversampling Technique (SMOTE) (21) and random methods, respectively. Thirty per cent of the unbalanced data is used as the test set, and the remainder as the training set, both of which are returned as a list. In addition to this, model tuning is required. The functions ‘mi_tune_rp’, ‘mi_tune_rg’ and ‘mi_tune_xgb’ use the original dataset to tune the parameter spaces of classification and regression tree (CART), random forest (RF) and extreme gradient boosting (XGBoost), respectively, and then draw the tuning stages plots and return them along with the tuner. Last, the functions ‘mi_train_rp’, ‘mi_train_rg’, ‘mi_train_xgb’ and ‘mi_train_BP’ train models with training sets for CART, RF, XGBoost and back propagation neural network (BPNN), respectively, and validate models with test sets to obtain the trained model and validation results. Finally, confusion matrices (CMs) are calculated and heat maps are plotted using the ‘mi_get_confusion’ and ‘mi_plot_heatmap’ functions. Furthermore, a custom wrapper function ‘mi’ is provided to streamline the implementation of steps of the MantaID workflow. In addition to quick large-scale ID identification based on machine learning approaches, MantaID offers a slower but more comprehensive ID recognition method based on online retrieval. This method covers 542 databases and can provide thorough small-scale ID recognition tasks and be used as a complementary method whenever the users want to, taking advantage of the up-to-date information available in the remote databases. For practical use, the aforementioned framework method has been implemented as an open-source R package called MantaID, and the steps of the construction of a MantaID model for ID identifications are described later.

### MantaID model

#### Data acquisition

MantaID searches public databases for and downloads ID datasets. At first, the function ‘mi_get_ID_attr’ is used to connect to the Ensembl database via the biomaRt package (22) and retrieve 3374 attributes of the human genome–related dataset in our test (23, 24). MantaID can be applied to other species datasets by modifying the argument ‘dataset’ of the ‘mi_get_ID_attr’ function and supports the use of all datasets listed in the R package biomaRt (22). After the retrieval of data, a filter routine based on regular expressions is implemented, leaving 68 ID-related attributes. Then, the attribute data frame is passed to the ‘mi_get_ID’ function, which returns the list of corresponding datasets from the Ensembl and rebuilds it into a long data frame, obtaining 2 936 411 rows. Twenty-nine datasets that lack ID information are eliminated by manual inspection. Finally, a data frame with ID and class columns and 2 751 478 rows is generated.

#### Data preprocessing

MantaID converts ID data into the format required by machine learning algorithms . The first step is to get the length of the longest ID using the ‘mi_get_padlen’ function. The ‘mi_split_col’ function then takes the length and the ID data frames as arguments, splits each ID element by character into a vector, fills the length to the maximum length and combines them by row, before returning a wide data frame containing the ID location information. The ‘mi_to_numer’ function then converts the input data frame features into computable numeric type features by constructing a mapping from characters to numbers.

#### Data balancing

MantaID balances the minority and majority classes in training datasets. A common method is the random sampling method, which balances the model by randomly selecting a minority class sample to add copies to it and a majority class sample to remove copies from it. The limitation of random sampling is that the model’s capacity to generalize may be compromised due to excessive sample duplication (25). Therefore, the SMOTE technique is also used for oversampling, whereas the random method is used for undersampling. The main advantage of using the SMOTE method is avoiding the overfitting caused by undersampling with the random method. MantaID balances data with the ‘mi_balance_data’ function, which takes as an input a data frame that contains unbalanced data, and then performs data balancing on it. Thirty per cent of the original balanced data is used as a test set, and the rest as a training set. The returned results from the function are formatted as a list. In addition to balancing the data, feature filtering is necessary for improving model accuracy when the datasets are typically noisy and contain a large number of irrelevant features.

#### Feature filtering

MantaID eliminates irrelevant and redundant features by estimating the feature covariance and Gini significance. Since the length of the longest ID determines the number of features included in the processed dataset, it is anticipated that there would be redundant features that need to be screened. Prior to filtering, the ‘mi_plot_cor’ function computes the Pearson correlation coefficient of the features to generate the covariance matrix and plots the heat map with its value as the color depth. Next, the ‘mi_get_importance’ function calculates Gini impurity to indicate the redundancy of the features, and a histogram is presented for it. Finally, low-weighted features are deleted using a threshold method based on covariance and importance. The filtered data are subsequently fed to the machine learning algorithms to generate classification models.

#### Model selection

MantaID contains four machine learning models for the large-scale and automatic identification of IDs: CART, RF, XGBoost and BPNN.

CART (26, 27) uses a tree structure to classify samples into different categories based on the distribution of features in a specific dimension of the samples. All the features and possible split points in the training set are traversed to find the best splitting feature and best split point. The training dataset is then split into two subsets using the best splitting feature and split point, with the results determined as the left and right subtrees, respectively, and the search is repeated for each subtree. The best splitting feature and best split point of each leaf node are determined repeatedly, allowing each leaf node to be partitioned into left and right subtrees. The pseudocode of the implemented algorithm in MantaID is given in Algorithm 1 (see the Supplementary File).

RF (28, 29) is based on bootstrapping using a small set of features to generate a large number of decision trees, which are then used to classify new data with greater accuracy than a single decision tree. The pseudocode of the RF algorithm is presented in Algorithm 2 (see the Supplementary File).

Based on the gradient boosting decision tree (30), XGBoost (31, 32) is an optimized distributed gradient boosting library that can massively parallelize the boosting tree. The main strength of using XGBoost is in continuously adding trees and performing feature splitting to grow. Each new tree is equivalent to learning a new function that fits the residuals of the previous one. When training is complete, we have *k* trees, each of which corresponds to a leaf node based on sample characteristics, and the score for each leaf node adds up to the sample’s prediction value. A detailed pseudocode is presented in Algorithm 3 (see the Supplementary File).

The learning process of BPNN is divided into two stages (33, 34): forward signal propagation and backward error propagation. When the actual output of the output layer does not match the desired output in the forward propagation process, the error advances to the backward propagation stage, obtaining the error signal of each unit as a basis for correcting the weights of each unit. The pseudocode of this process is shown in Algorithm 4 (see the Supplementary File).

#### Model tuning

MantaID uses the hyperband approach to tune hyperparameters for CART, RF and XGBoost before training. Hyperband (35), as an extension of Successive Halving (36), is used to determine the optimized setting of operational parameters. For each set of parameter combinations, the loss value is computed using R package ‘mlr3hyperband’ (37). Following the evaluation of the loss of each parameter combination, only one-third of parameter combinations with the lowest loss values are selected for the next iteration. The aforementioned process is summarized in the pseudocode form in Algorithm 5 (see the Supplementary File).

Parameter configurations for BPNN are tuned using a different approach as follows. BPNN consists of a four-layer fully connected network with an input layer, two hidden layers and an output layer. First, the number of nodes in the input and output layers is set equal to the number of features and categories, while the number of nodes in the hidden layer is fixed at 40 according to some rules of thumb that have been previously described (38). Next, Rectified Linear Unit (Relu) is used as the activation function for the hidden layer instead of sigmoid and tanh because it is less computationally intensive and does not tend to saturate, while Softmax is used for the output layer. Finally, the Adam (39) optimizer is implemented to compute individual adaptive learning rates for different parameters, circumventing the need for hyperparameters tuning. The aforementioned process is described in Algorithm 4 (see the Supplementary File).

#### Model training

Balanced datasets are used for training. To begin the process, the training and test sets are accepted as parameters by functions ‘mi_train_rp’, ‘mi_train_rg’ and ‘mi_train_xgb’ in order to train and validate CART, RF and XGBoost models. After the CMs of the validating results are calculated and plotted as heat maps, trained models are returned as a list. For BPNN, the ‘mi_train_BP’ function sets epoch and batch size first equal to 64 and the batch size equal to 128, based on the empirical guidelines in the literature (38), and it also accepts the training and test sets as inputs. Likewise, after training is complete, the CM is returned and plotted as a heat map.

#### Model unification and scoring

**Figure 2.**
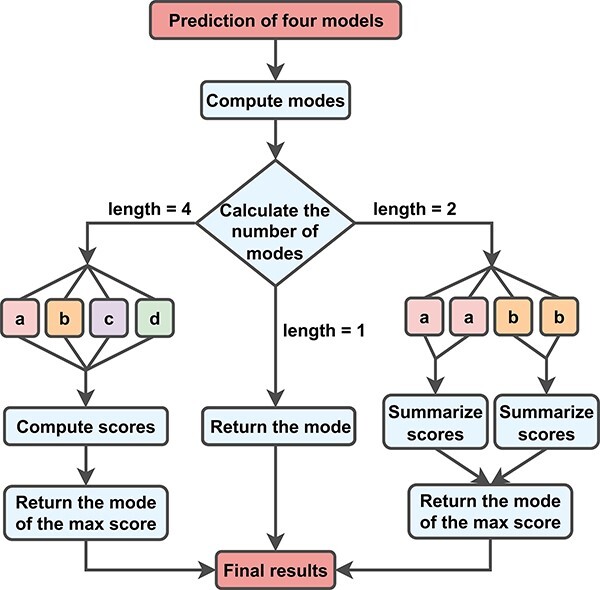
New metrics for aggregating MantaID models. To incorporate the information, we multiply the model’s F1 score metrics by the mismatch rates of other models to calculate the submodel’s score. When the submodels disagree, we assign a score to each result and select the best one.


The use of an even number of models makes it impossible to directly derive the final result using the voting method. To resolve this issue, we present a new method for aggregating models, as depicted in Figure 2 and as follows. MantaID uses the voting method when there is a majority class in prediction results; however, when there are scattered opinions, MantaID uses the following scoring formula for evaluation:

(1) $${N_{{score}}}{\; = \;F1}_x^{N}\mathop \prod \nolimits_{R \in \neg {N};\;y = {val}\left( R \right)} \left( {c + \left( {1 - c} \right) \cdot P{{\left( {y|x} \right)}^R}} \right){\rm{\;}}$$

(2) $$\frac{\partial {Score}_{N}}{{\partial P{{\left( {y|x} \right)}^{\neg {N}}}}}{\rm{\; = \;}}1 - c$$

where ${N_{{score}}}$ is the score of model ${N}$, ${F1}_x^{N}$ is the F1 score of model ${N}$ for category $x$, val(*R*) is the prediction result of model ${N}$, *P*(*y*/*x*)^N^ is the probability that model ${N}$ misclassifies $y$ as $x$ and $c$ is a constant value that determines the degree of influence of other models on the score of the current model. The larger the $c$ value, the lower the bias derivative $\frac{\partial {Score}_{N}}{{\partial P{{\left( {y|x} \right)}^{{\neg N}}}}}$ and the smaller the effect, according to Equation (2).

Although accuracy is a good indicator of the model’s correct prediction rate of random individuals, it works poorly on unbalanced datasets and is inclined to hide serious classification errors for classes with few samples (40). This problem can be avoided by using F1 score, which is a good balance between accuracy and implementability, reflecting the model’s effectiveness in classifying this class (41); therefore, this evaluation criterion in MantaID is implemented based on the F1 score. In addition, to fully utilize the existing information, we add other models’ misclassification rates when computing a model’s score, in order to avoid being biased in the evaluation. Finally, the model with the highest score (${N_{{\rm{score}}}}$) is selected and is then evaluated by recall, precision, accuracy and the F1 score. For convenience, we use the following abbreviations: TP, true positive; FP, false positive; TN, true negative; FN, false negative; Acc, accuracy; Pre, precision; Rec, recall; and F1, F1 score.


(3) $${\rm{Acc\, = \,}}\frac{{{\rm{TP + TN}}}}{{{\rm{TP + FN + FP + TN}}}}$$

(4) $${\rm{Pre\, = \,}}\frac{{{\rm{TP}}}}{{{\rm{TP + FN}}}}$$

(5) $${\rm{Rec\, = \,}}\frac{{{\rm{TP}}}}{{{\rm{TP + FP}}}}$$

(6) $${\rm{\,F1 = \,}}\frac{{{\rm{2TP}}}}{{{\rm{2TP + FN + FP}}}}$$

### MantaID web application

MantaID includes a user-friendly web application for ID identification, which is available free from the website at https://molaison.shinyapps.io/MantaID/. The primary MantaID interface features a search box that lets you input your query and implement the ID identification methods available in MantaID. A more comprehensive, crawler-based algorithm is also adopted by the MantaID web application to improve the accuracy of the ID identification. First, MantaID performs pattern matching with regular expressions obtained from identifiers.org hosted by European Bioinformatics Institute (42) to filter out missing or malformed data. Second, MantaID connects to the Uniform Resource Locators (URLs) of IDs using the ‘httr’ R package (43). An ID is determined as non-existent or inaccessible when the connection yields an error Hypertext Transfer Protocol (HTTP) status code, such as the 404 page-not-found error. Finally, MantaID retrieves and analyzes the text from the database webpages to determine whether an ID does not exist based on the presence of contextual keywords such as ‘failure’ or ‘No correct information’. These steps should be sufficient for determining the existence of IDs and the databases to which they belong, excluding invalid IDs.

To assist new users, example queries and guidelines are provided alongside the search box. As the identification process progresses, each successfully matched database name and pertinent information are returned as a row in the result table, displayed beneath the search box and can be saved and outputted in various file formats. The original names retrieved from the databases are added with modifiers and shown in the same column as ‘name’ to distinguish between the identical entities within databases, enabling an ID query to identify all matched biological entities such as a gene, protein, or transcript (44, 45).

The advanced search option is also provided: (i) the user can specify the maximum time for accessing each entry, (ii) the user can select whether to go directly to the associated database using the provided URL, (iii) the user can specify the type of object indicated by the ID and (iv) the user can select between local (intensified) and global (diversified) search strategies. A batch search tool is supplied to implement the described MantaID methodology for large quantities of unidentifiable ID data files. The batch search results can be formatted and aligned, and data can be outputted for download in a variety of user-specified formats, as well as for reproducing the model predictions.

## Results

### Performance evaluation of the MantaID Model

**Figure 3.**
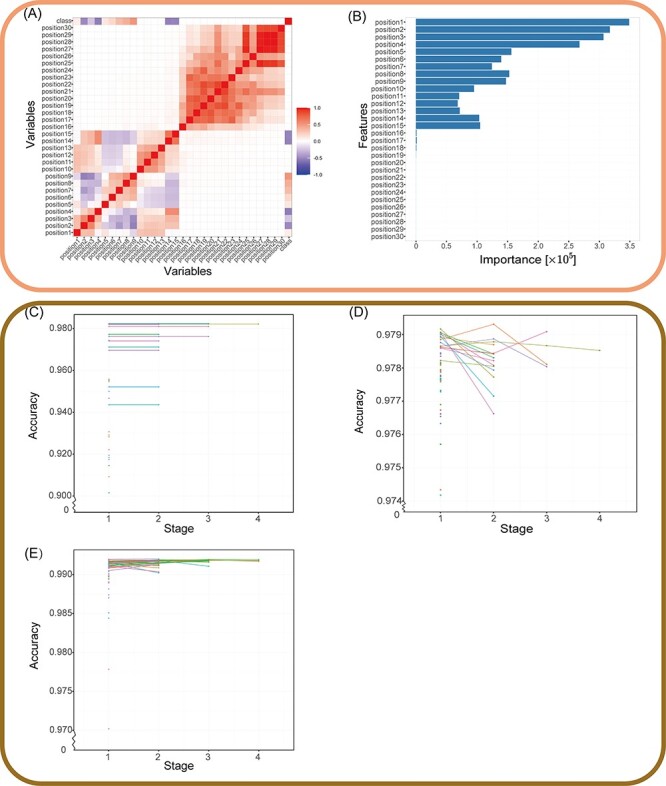
Validation of the MantaID model performance. (A and B) The result of features selection. (A) Correlation heat map. Positive values mean positive correlation; negative values mean negative correlation, as evaluated by Pearson's correlation test. (B) Features importance computed by RF. The horizontal coordinate is the Gini impurity, an indicator for evaluating importance, and the vertical coordinate is the feature. Stage plot for Hyperband tuning of (C) CART, (D) RF and (E) XGBoost. Each line or point represents a set of related parameters, and Hyperband algorithm discards configurations with a percentage of ${\rm{\;}}\frac{1}{\eta }$ to cut training time. Notably, the cart model’s polygon line appears to be stagnating as a result of the minimal accuracy change between stages when compared to the span.

We evaluated MantaID on datasets assembled from public databases to demonstrate its superior ability to identify IDs. MantaID was executed to construct an ID identification model using 39 datasets (Table 2). After the data processing steps were completed, the correlation heat map and importance histogram were generated based on the feature covariance matrix and the feature selection results. As shown in Figure 3, the posterior 10 features have low feature importance and low relevance with the target class, which supports our hypothesis that the redundancy is caused by padding IDs; thus, these features were regarded as redundant and deactivated.

**Table 2.** Databases and datasets currently available on MantaID model

Name	Imbalanced	Balanced	Description
The Consensus CDS	32 717	60 736	CCDS ID
Conserved Domain Database	7204	68 390	CDD ID
ChEMBL	4030	69 342	ChEMBL ID
EMBL	199 350	139 545	European Nucleotide Archive ID
Ensembl exon	852 763	596 934	Exon stable ID
Ensembl gene	68 016	50 146	Gene stable ID
Entrez Gene Database	22 927	63 673	NCBI gene (formerly Entrezgene) ID
HAMAP	358	70 444	HAMAP ID
HGNC	39 780	58 617	HGNC ID
HGNC Transcript	232 496	162 747	Transcript name ID
PANTHER	23 775	63 418	PANTHER ID
Interpro	17 612	65 267	Interpro ID
Merops	780	70 317	MEROPS—the Peptidase Database ID
miRBase	1846	69 997	miRBase ID
Protein Data Bank	48 239	56 079	PDB ID
Pfam	6595	68 572	Pfam ID
pfScan	895	70 282	PROSITE profiles ID
PIRSF	949	70 266	PIRSF ID
PRINTS	1483	70 106	Prints ID
Protein	490 333	343 233	INSDC protein ID
Reactome	2495	69 802	Reactome gene ID
Refseq mrna	62 046	51 937	RefSeq mRNA ID
Refseq ncrna	15 828	65 803	RefSeq ncRNA ID
Refseq peptide	57 215	53 386	RefSeq peptide ID
Rfam	58	70 534	RFAM ID
Rfam transcript	1461	70 113	RFAM transcript name ID
RNAcentral	89 729	62 810	RNAcentral ID
ScanProsite	881	70 287	PROSITE patterns ID
Structure–Function Linkage Database	64	70 532	SFLD ID
SMART	1020	70 245	SMART ID
SUPERFAMILY	1113	70 217	Superfamily ID
TIGRFAMs	594	70 373	TIGRFAM ID
UCSC	226 788	158 752	UCSC Stable ID
UniProt Archive	90 791	63 554	UniParc ID
Uniprot gene	20 438	64 420	UniProtKB Gene Name symbol
Uniprot isoform	24 825	63 104	UniProtKB isoform ID
Uniprot TrEMBL	61 771	52 020	UniProtKB/TrEMBL ID
Uniprot Swiss-prot	19 287	64 765	UniProtKB/Swiss-Prot ID
WikiGene	22 926	63 673	WikiGene name

Then, the ratio of the largest majority class to the smallest minority class was used to measure the imbalance degree. According to Table 2, the ratio for the original dataset is about 14 702:1, indicating that the data are extremely imbalanced. After completing the data balancing steps, the ratio is reduced to approximately 12:1, suggesting that the data imbalance is significantly reduced. After balancing the data, the three models of CART, RF and XGBoost were tuned using Hyperband methods, with${\rm{\;}}\eta $ set to 3, leaving only one-third of the possible hyperparameter combinations for each of the four stages. In total, 49 parameter combinations were tuned for all stages in the parameter spaces of the three models, as shown in Table 3. The results of the parameter tuning for all stages are shown in Figure 3. The parameter combination with the lowest loss value in the fourth stage was regarded the most robust and was chosen for each model.

**Table 3.** Parameter configuration for CART, RF, XGBoost and BPNN

Model	Classification Tree	RF	XGBoost	Back Propagation
Complexity parameter	0.00053			
Maximum depth of tree	24	368	8	
Minimum observations in a node	4			
Number of cross-validations	0			
Number of competitor splits retained	3			
Number of decision trees		385		
Criteria for fragmentation		‘gini’		
Minprop		0.017		
Evaluation with the off-bag sample		TRUE		
Importance		‘impurity’		
Eta			0.29	
Regularization factor		0.014		
Proportion of random sampling			0.84	
Iterative model			‘gbtree’	
Minimum loss function descent value			0	
Regularization term of weight			0.92	
Number of passes			10	
Column sampling			0.99	
Iterations				64
Proportion of training set as the test set				0.3
Loss function				‘Categorical_crossentropy’
Number of samples per workout				128
Optimizer				‘Adam’

The MantaID model uses Hyperband to tune the parameters of the first three algorithms

**Figure 4.**
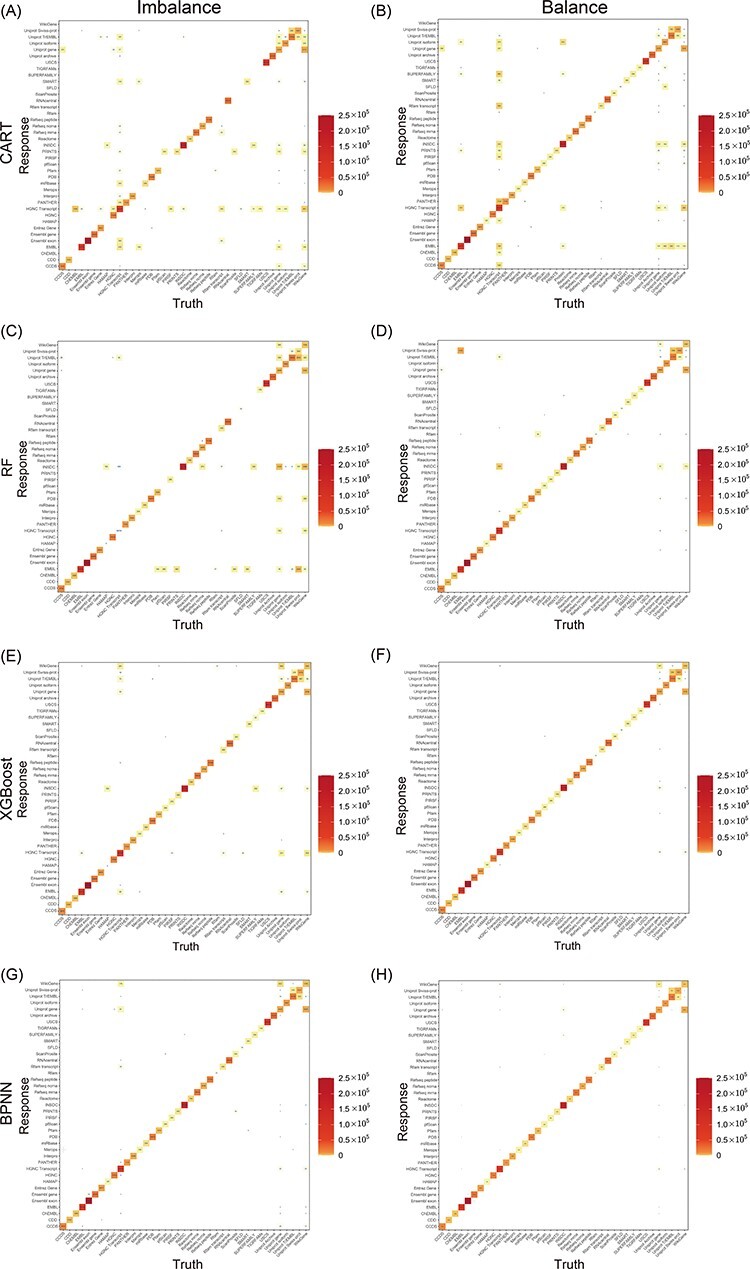
Heat maps of the CMs for models. CART, RF, XGBoost and BPNN, which were trained on both balanced and unbalanced data, are included. The number of truth-prediction pairs is shown by the value in the box. The more the model is accurate, the more the values are concentrated on the diagonal. Through comparing models with and without balancing samples, we discovered that while accuracy did not noticeably improve as a result of balancing datasets, the models performed better for minor classes.

Next, the balance effect was assessed by training the model with the optimal set of parameters using both the balanced and unbalanced training datasets. The assessment results were presented as heat maps representing the CMs (Figure 4). The diagonal numbers in the CMs were used to compare models trained on the balanced and unbalanced datasets, because a change in the model’s specificity was a better outcome measure for qualifying the results of minority classes in both the balanced and unbalanced datasets than the overall accuracy. Our results show that, before balancing, CART and RF misclassified nearly all minority classes, XGBoost misclassified only a few minority classes and BPNN correctly classified almost all the minority classes. After balancing, all the four models almost perfectly classified the minority classes, indicating that MantaID effectively constructed a robust classifier when learning from a large quantity of unbalanced ID datasets.

Finally, the performances of our models were compared by using accuracy, precision, recall and F1 scores, as summarized in Table 4. The high recall rates for most ID classes provide confidence for the accurate classifications. However, low precision values were obtained for some minority classes, which is due to the incorrect classification of a small portion of a large number of majority classes into minority classes. Most models failed to accurately predict WikiGene IDs, due to the fact that WikiGene (46) unites multiple data sources, such as UniProt and Entrez, containing overlapping information. What stands out in Table 4 is that the results of integrated model were superior to those of the individual models in almost every category, indicating that the integrated model inherits the advantages of individual models.

**Table 4.** Accuracy, precision, recall and F1 score

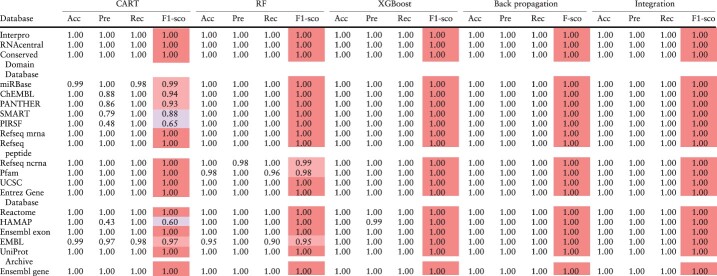
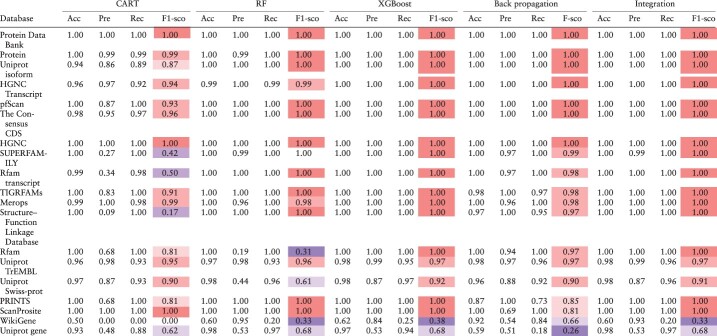

The evaluation of the performance of the CART, RF, XGBoost, and BPNN models was conducted based on the scores presented in the table. The scores were used to integrate the outcomes of the models that were applied to the balanced data. Higher F1 score values reflect better performance. Accuracy, precision, recall and F1 score are represented in the table as Acc, Pre, Rec and F1-sco, respectively

### Features of MantaID web application

**Figure 5.**
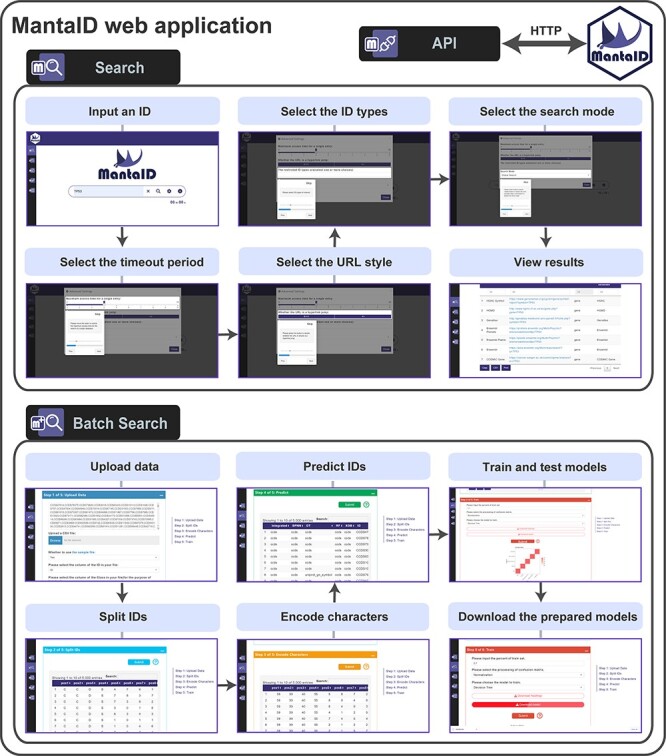
The features of the MantaID web application. The setting panels allow users to configure the basic and advanced settings; basic settings populate settings panels by default, whereas advanced settings enable a more granular control.

MantaID functions can be used directly via the MantaID’s Shiny application in an easy and reliable way. ManatID contains three main modules (Figure 5): (i) a general search engine; (ii) a more advanced search engine, named as the batch search tool, and (iii) a fully documented API.

A Google-like search engine is provided to allow users to make queries on IDs easily and reliably. ID identification can be carried out across all existing biological databases listed on (identifiers.org) using default, or customizable with advanced options to perform advanced crawler-based, personalized algorithms when the user has partial knowledge or imperfect information about the sources of the unknown IDs.

The batch search tool of MantaID shiny app provides a template comprising five steps to facilitate the large-scale ID identification, as well as guideline and extensive helps for customizing parameters of the MantaID model to pursue better identification efficiency. All results can be aggregated into a single table displayed and can be outputted into various formats for ease of analysis.

The API is provided for interfacing with other applications or tools and allows us to integrate the services provided by MantaID into other workflows. This paves the way for other applications to integrate ID identification into their data processing pipelines.

The advantages of using MantaID shiny app are manifold: (i) it is cost free, platform =-independent, user-friendly and available to any internet-connected user; (ii) it can perform all the methodologies and methods available in MantaID and (iii) user interactions can be restricted to circumvent undesired modifications.

## Discussion

In this work, the MantaID was developed based on machine learning approaches to conduct the large-scale identification of unknown and heterogeneous IDs. Besides achieving a good level of accuracy, MantaID can predict thousands of IDs in a few seconds, e.g. in our test, 100 000 of IDs generated by randomly sampling the available ID datasets can be identified in 71 s.

Previous studies created ID mapping by formulating knowledge-based rules based on their understanding of mappings provided by selected databases (14, 15, 17, 18, 47, 48). These tools rely on metadata and annotations provided by databases to link IDs from different databases (49). Common database IDs, such as Ensembl (13) and RefSeq (50), serve as bridges between databases that lack direct linking of the same entity. The linkages were used as ID mappings that must be frequently updated, such as in the case of a recently published tool TogoID (17), which is dependent on manual curation and is updated every 2 weeks. Lack of frequent updating can result in query failures for new IDs. For example, UniProt (14) can support 98 databases for conversion, DAVID (15) only supports 41 databases for conversion and TogoID (17) only supports 48 databases for conversions. In contrast, MantaID employs a series of machine learning models trained based on a large number of database IDs; once the MantaID model derives the rules of ID to database mapping automatically from the training datasets, it uses the automatically generated ID database mapping to perform ID interpretation. Therefore, MantaID does not require human intervention for updates. In addition, the IDs are not unique across different databases and there is no universal agreement on the composition of a database ID, i.e. an artificial, fictitious ID created for testing purpose could pass as a real ID in some databases. Tools present in the literature (15, 17, 18) have quite limited ID conversion capabilities that are primarily dependent on ID and database mappings created by annotations. The ID mappings in these tools are fixed and can only be modified by tool’s maintainers, necessitating a stringent ID validation prior to ID conversions (49). Therefore, these tools can only accept input of IDs specified within their ID-to-database mapping tables. In contrast, MantaID is a machine learning–based tool that can interpolate and impute any IDs supplied by users based on principles derived from probabilistic models. MantaID aims to identify all IDs of existing biological databases; MantaID models are built and trained on a vast amount of data from a variety of databases, so it is possible to find a legitimate use for an ID that was previously thought to be fictitious. We believe that the MantaID approach is better suited for dealing with a growing number of databases, as it generates ID-to-database mappings automatically without the need for human annotation or intervention.

MantaID is a novel hybrid approach combining machine learning–based algorithms and expressive power of regular expressions to capture the variability during the process of ID matching. Regular expressions are a general-purpose string-matching technique that can only be used to expedite the identification of IDs when ID names are constructed according to carefully and precisely defined rules. However, there are no standard rules set for constructing ID names and the ID names can be similar across databases; in most cases, according to our experience, the same regular expressions can match multiple IDs from different databases. For example, on https://identifiers.org/ (42), the same regular expression pattern ‘^[A-Z0-9]+$’ that is defined for Catalogue of Somatic Mutations in Cancer Gene, Bacterial Tyrosine Kinase and DEPhOsphorylation databases can also match the ChEMBL database IDs (with a regexes of ‘^CHEMBL\d+$’). The inefficiency of regular expressions has been encountered and noted in the literature (51, 52). In addition, overly formulated complex regular expressions for ID identifications can exhibit catastrophic backtracking, consuming the majority of the computer’s computing power (53–55). So regular expressions alone are not sufficient enough for identifications of IDs that can be inconsistently or erratically formulated in many databases. On the other hand, besides the use of regular expressions for a global, coarse-grained identification of IDs, MantaID employs machine learning approaches to identify IDs in order to achieve high efficiency and effectiveness. MantaID generates data-driven, recursive models that can be automatically trained and improved by adding more datasets.

MantaID can identify IDs without requiring explicit knowledge of database names, which, to our knowledge, is a functionality that none of other tools provide (56, 57). This functionality is expected to facilitate the automation of the data-driven analysis pipelines that involves the translation of unsorted free text words extracted from research papers containing IDs of different fields into biologically relevant information via databases. For example, it has been a difficult task to construct a genome-scale metabolic model that involves merging and processing of various omics data, which are managed by different databases using different IDs (58), into a structured and unified model; it has always required human knowledge of the databases from which the IDs originate, in order to translate and search the IDs using databases, due to the lack of software tools capable of automatically identifying the ID database (59, 60). Now, with the help of MantaID, large amounts of free text in literature can be fed into MantaID to search for their meanings in databases, and based on the organized ID meaning tables, protein interactions, gene–disease associations, etc. can be constructed (61–64).

## Conclusion

In summary, MantaID is capable of identifying IDs rapidly and is based on various machine learning approaches that are tailored for high accuracy and efficiency. Due to the data-driven nature of our proposed framework approach, MantaID supports the identification of all types of IDs across diverse databases, thereby avoiding the limitations encountered by a few other ID conversion programs By eliminating the need to manually look up biological IDs in online databases, it is envisioned that MantaID will become an indispensable tool for the creation of large-scale models by assimilating and integrating large quantities of ID data linking all biological knowledge.

## Supplementary Material

baad028_Supp

## Data Availability

The source files and instruction of API are contained within the R package (https://bitbucket.org/Molaison/mantaid/src/main/).
