# Supplementary material for: MantaID: a machine learning–based tool to automate the identification of biological database IDs
Source: Database (Oxford). 2023 May 9;2023:baad028. doi: 10.1093/database/baad028 (PMC10168000; doi:10.1093/database/baad028)
Supplement: baad028_Supp [file baad028_supp.zip › suppl_data/Supplementary File.docx]

**
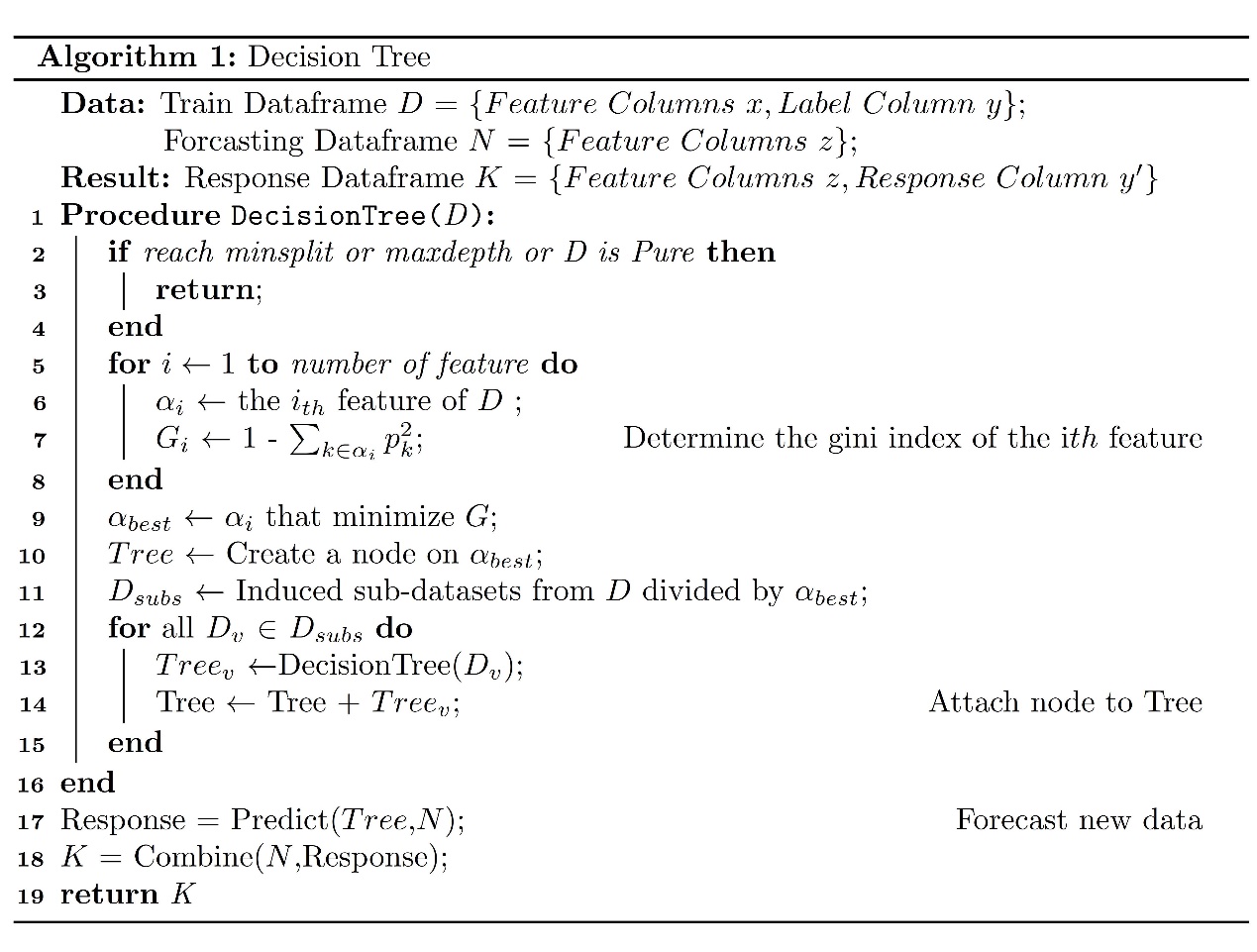
Algorithm 1: Pseudocode of decision tree model used in MantaID.**


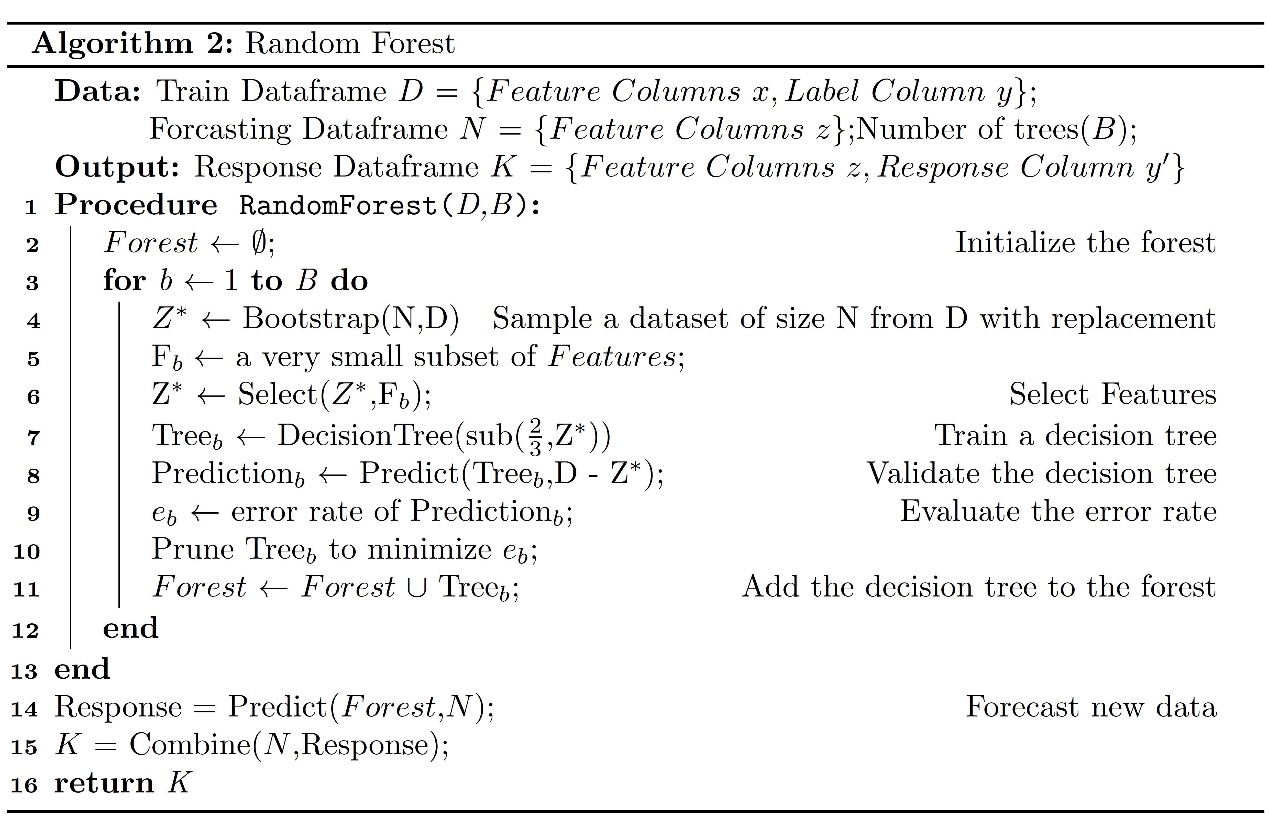
**Algorithm 2: Pseudocode of random forest model used in MantaID.**

**
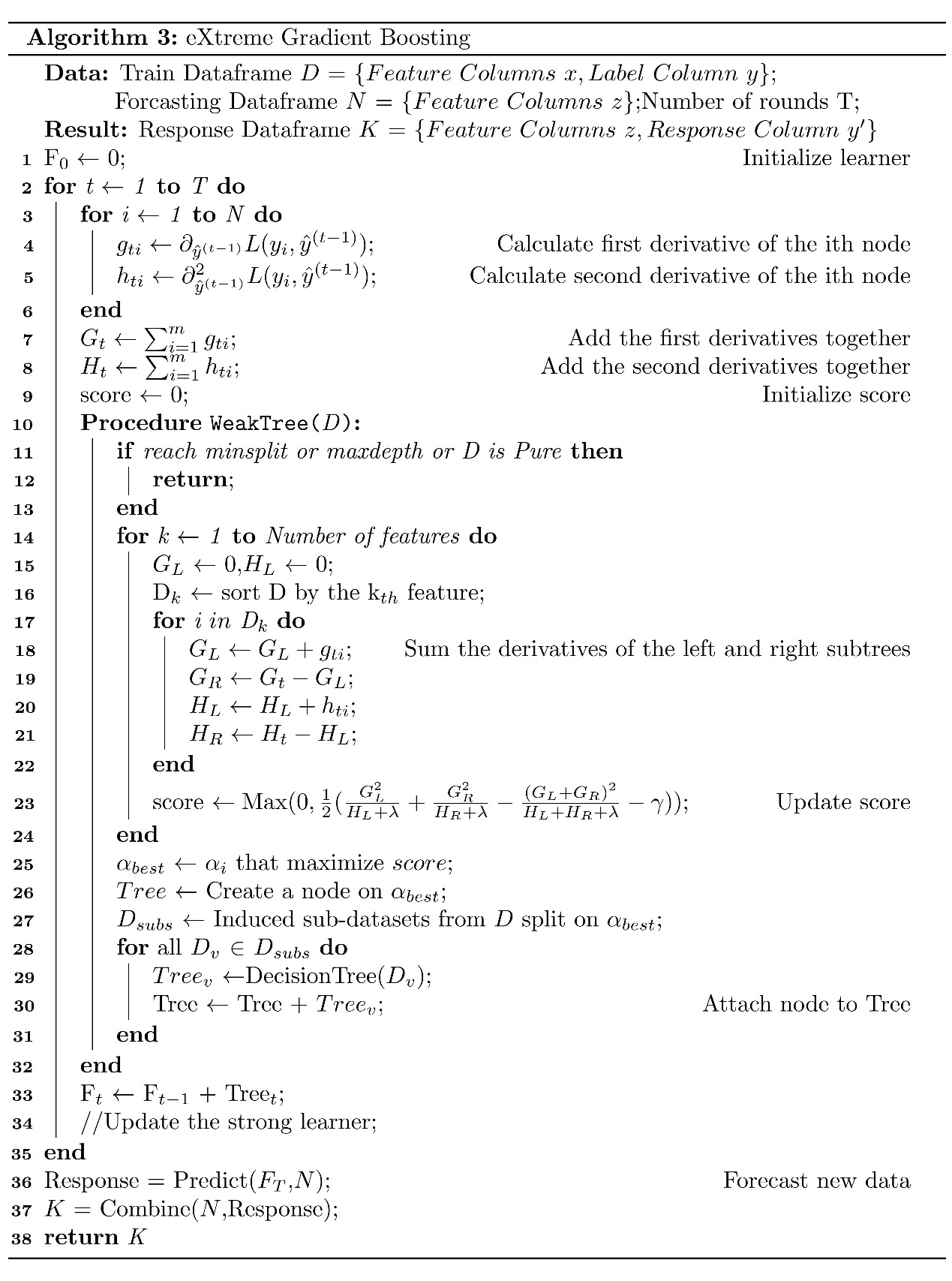
Algorithm 3: Pseudocode of extreme gradient boosting model used in MantaID.**

**Algorithm 4: Pseudocode of back propagation neural network model used in MantaID.** **
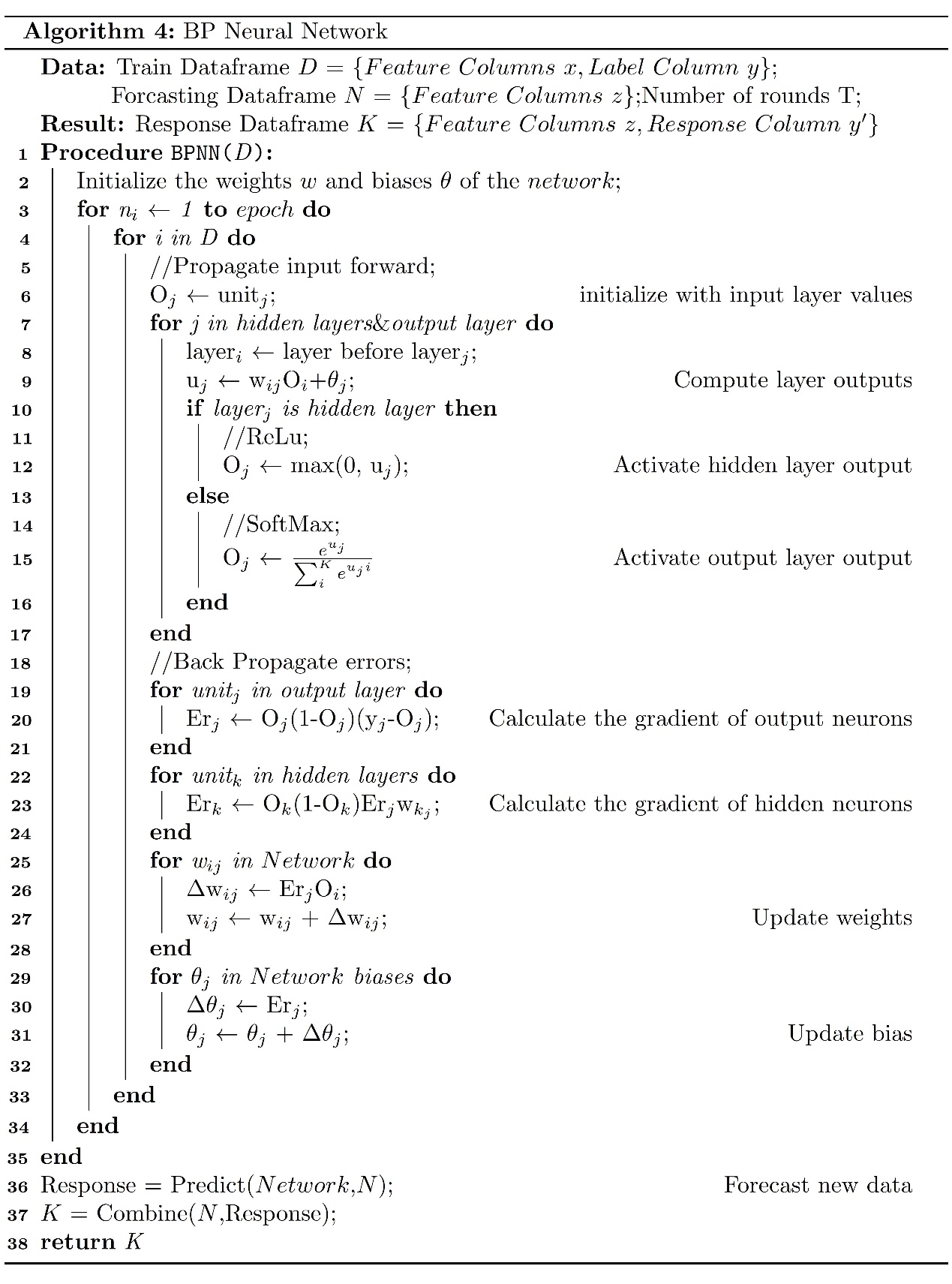
**

**Algorithm 5: Pseudocode of Hyperband optimization used in MantaID.** **
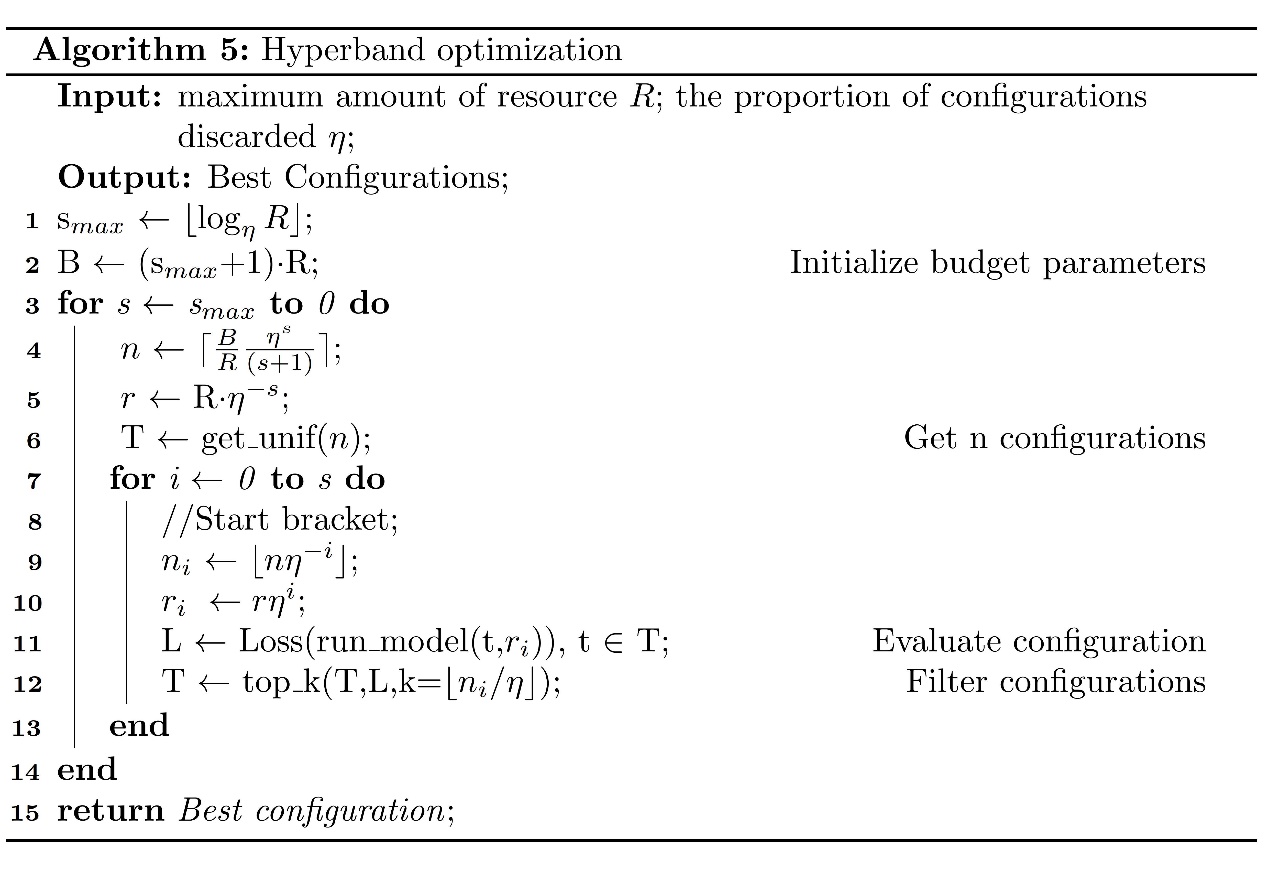
**
